# Spinal cord injury induces acute microbiome shock and system‐wide transcriptomic reprogramming

**DOI:** 10.1002/imt2.70128

**Published:** 2026-05-03

**Authors:** Chi Zhang, Yufei Du, Mingxin Wu, Chuang Li, Ruizhi Jiang, Enlin Qi, Shaolong Li, Xianfu Yi, Bo Chu, Shiqing Feng, Hengxing Zhou

**Affiliations:** ^1^ Department of Orthopaedics The Second Qilu Hospital of Shandong University, Shandong University Centre for Orthopaedics, Cheeloo College of Medicine, Shandong University Jinan China; ^2^ Department of Endocrinology and Metabolism The Second Qilu Hospital of Shandong University, Cheeloo College of Medicine, Shandong University Jinan China; ^3^ Department of Spine Osteopathia The First Affiliated Hospital of Guangxi Medical University Nanning China; ^4^ Department of Orthopaedics Qilu Hospital of Shandong University, Shandong University Centre for Orthopaedics, Advanced Medical Research Institute, Cheeloo College of Medicine, Shandong University Jinan China; ^5^ Department of Bioinformatics, School of Basic Medical Sciences Tianjin Medical University Tianjin China; ^6^ Department of Cell Biology, School of Basic Medical Sciences, Cheeloo College of Medicine Shandong University Jinan China

## Abstract

This study investigates the systemic consequences of spinal cord injury (SCI), with a particular focus on alterations in the gut microbiome and multi‐organ transcriptomic responses. We identify a rapid and severe disruption of the gut microbiota—termed “microbiome shock”—that emerges within 12 h post‐SCI and persists before gradually resolving by 5 days post‐injury. To support further research in this field, we established an open‐access resource, the Spinal Cord Injury Gut Microbiome and Multi‐Organ Gene Expression Atlas (SCIGAMA).

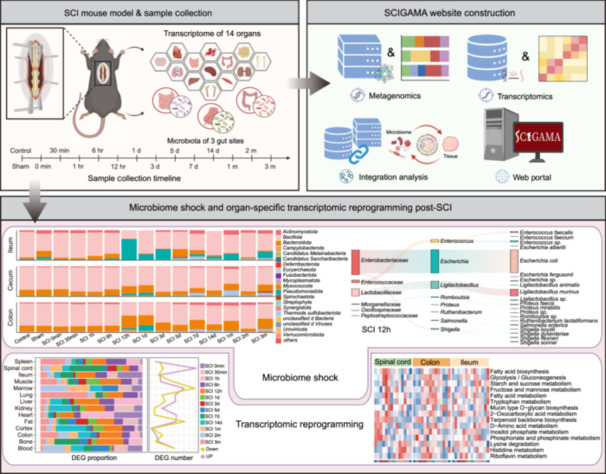

To the editor,

Spinal cord injury (SCI) represents a severe and often life‐altering disruption of the central nervous system that causes substantial disability and mortality. Most cases are caused by traumatic events such as traffic accidents and falls [1], with over 15.4 million individuals affected worldwide as of 2021 [2]. Beyond spinal cord damage, SCI induces systemic dysfunctions involving multiple organs, disrupting whole‐body homeostasis [3] and increasing the risk of neuropathic pain, cardiovascular disease, bladder cancer, and chronic bone marrow failure [3, 4]. These systemic consequences underscore the need to study SCI beyond a spinal cord‐centric perspective.

The gut microbiome has increasingly been recognized as a crucial modulator regulating immunity and metabolism [5, 6]. Dysbiosis is linked to disease progression and may offer therapeutic targets. SCI‐induced secondary pathologies, including neurogenic bowel dysfunction, significantly alter the gut microbiota [7]. While some studies have explored post‐SCI gut microbiota changes longitudinally [8, 9], many prior investigations relied on single time‐point analyses [10, 11], missing temporal dynamics. Clarifying how these processes evolve over time is fundamental to defining their contribution to SCI pathophysiology and therapeutic relevance.

Transcriptomic profiling provides valuable insights into the molecular mechanisms underlying SCI. Through genome‐wide transcriptomic profiling, key spatial and temporal features of SCI have been partially characterized [12, 13]. However, a systematic, time‐resolved characterization of transcriptomic changes across multiple organ systems remains limited, hindering our understanding of SCI pathology and its systemic effects.

To address this gap, we analyzed the temporal dynamics of the gut microbiome and multi‐organ transcriptomes following SCI. We identified “microbiome shock”, marked by acute microbial disruption at 12 h and partial recovery at 5 days post‐SCI. These changes were accompanied by widespread transcriptional reprogramming in various organs. Through integrative analyses across time points and tissues, we uncovered the dynamics of systemic inflammation and metabolic remodeling induced by SCI. These findings offer a systems‐level understanding of SCI pathogenesis and are integrated into the Spinal Cord Injury Gut Microbiome and Multi‐Organ Gene Expression Atlas (SCIGAMA), an open‐access resource for advancing SCI research.

## SPINAL CORD INJURY INDUCES ACUTE “MICROBIOME SHOCK”

**FIGURE 1:**
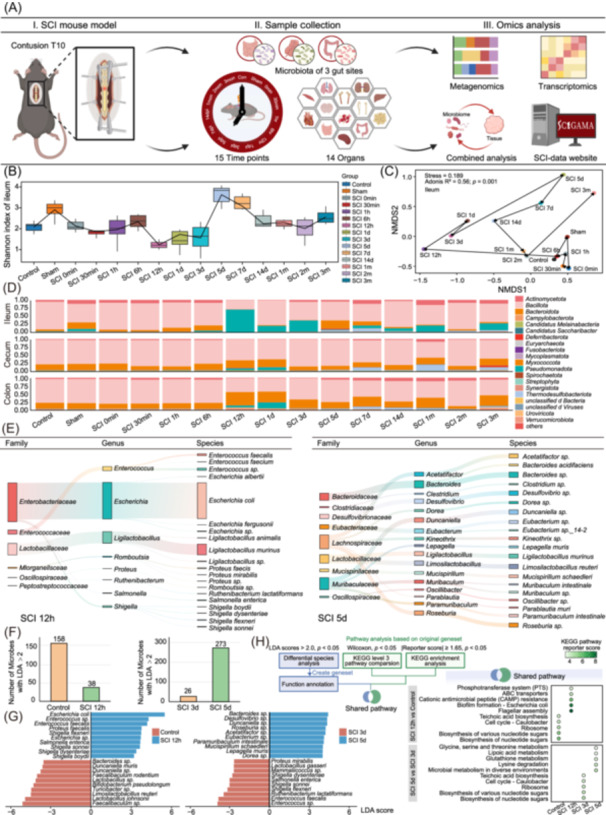
Spinal cord injury elicits an acute microbiome shock at 12 h post‐SCI, with microbial community restructuring commencing beyond 5 days. (A) Experimental design and multi‐omics workflow. (B) Temporal α‐diversity changes in ileal microbiota. (C) β‐diversity of ileal microbiota across time points. (D) Phylum‐level microbial composition in different intestinal segments. (E) Microbial community changes at 12 h and 5 days post spinal cord injury (SCI) shown by Sankey diagrams. (F) Numbers of highly abundant taxa between Control and 12 h post‐SCI, and between 3 days post‐SCI and 5 days post‐SCI. (G) Top 10 of differentially abundant taxa identified between Control and 12 h post‐SCI, and between 3 days post‐SCI and 5 days post‐SCI. Significant differences of these specific taxa were further validated by one‐way permutation test (9999 resamples, *p*. adj < 0.05). (H) Functional analysis of microbiota comparing Control and 12 h post‐SCI, and comparing 3 days post‐SCI and 5 days post‐SCI.

To delineate the systemic impact of SCI, we conducted a longitudinal analysis of the gut microbiome and multi‐organ transcriptomes. Metagenomic sequencing of the ileum, cecum, and colon was conducted across 15 time points from 0 minute to 3 months post‐SCI, alongside transcriptome profiling of 14 organs (Figure 1A). Significant temporal fluctuations in microbial α‐diversity were observed after SCI (Figure 1B, Figure S1A–C). Principal coordinates analysis and Non‐metric Multidimensional Scaling analyses further revealed distinct microbial trajectories at species‐level, although cecal communities showed minimal changes during the acute phase (within 48 h post‐SCI) (Figure 1C, Figure S1D–F). At the phylum level, the ileal microbiota underwent a profound shift at 12 h post‐SCI. The acute depletion of resident commensals led to a transient proportional dominance of *Pseudomonadota* (a phylum of opportunistic pathogens) during the acute phase (Figure 1D and Table S1). We term this acute, transient collapse of microbial diversity and expansion of pathobionts following SCI as “microbiome shock”, analogous to spinal shock− the transient loss of reflex activity below the level of acute spinal cord disconnection. At 12 h, *Escherichia coli* dominated, but by 5 days post‐SCI, the community markedly restructured, with a predominance of taxa from the families *Lachnospiraceae* and *Muribaculaceae*, including *Bacteroides acidifaciens*, and *Mucispirillum schaedleri*, which are all involved in metabolic [14] and immune homeostasis [15, 16] (Figure 1E). In contrast, communities in the Control, Sham, and SCI 0 min groups exhibited minimal differences, with 16 taxa being present in at least two groups (Figure S1G–J). To identify specific microbial taxa, we employed Linear Discriminant Analysis Effect Size (LEfSe) to compare differentially abundant taxa between the Control and SCI 12 h groups, and between the SCI 3 d and SCI 5 d groups. LEfSe revealed 38 differentially abundant taxa in the SCI 12 h group and 273 in the SCI 5 d group (Figure 1F). Opportunistic pathogens (*Escherichia coli*, *Enterococcus* sp.) were enriched at 12 h, whereas commensal genera (*Bacteroides* sp., *Duncaniella* sp., and *Desulfovibrio* sp.) predominated at 5 days post‐SCI (Figure 1G). Functionally, microbiota at 12 h post‐SCI were enriched in cationic antimicrobial peptide (CAMP) resistance, biofilm formation, and flagellar assembly, indicating immune evasion and increased motility. By 5 days post‐SCI, functional enrichment shifted toward metabolic pathways, reflecting metabolic reprogramming and partial recovery from microbiome shock (Figure 1H and Table S2).

We further investigated how the gut microbiota evolved over a more extended timescale. The number of differentially abundant taxa peaked at 14 days post‐SCI in the ileum and cecum, with long‐term changes persisting up to 3 months (Figure S2A–C). *Lactobacillus acidophilus*, a common probiotic, decreased sharply by 12 h, suggesting that early supplementation may be beneficial (Figure S2D). *Bacteroides thetaiotaomicron* increased and remained elevated for up to 3 months (Figure S2E). Functional annotation revealed metabolic activation in *Alistipes* sp., a genus elevated in Alzheimer's patients. In contrast, *Muribaculum* sp., a putatively probiotic species, exhibited widespread functional suppression following SCI. Similarly, *Bacteroides* sp., previously reported to be reduced in the fecal microbiota of individuals with autism spectrum disorder, also showed inhibition of various related functions after SCI (Figure S2F). These findings delineate the dynamic remodeling of the gut microbiota after SCI, featuring an acute phase of microbiome shock followed by a partial recovery phase characterized by the expansion of homeostatic symbiotic taxa.

## GUT MICROBIOME AND MULTI‐ORGAN TRANSCRIPTOME INTEGRATION REVEALS THE NECESSITY FOR PHASE‐SPECIFIC INTERVENTIONS POST‐SCI

**FIGURE 2:**
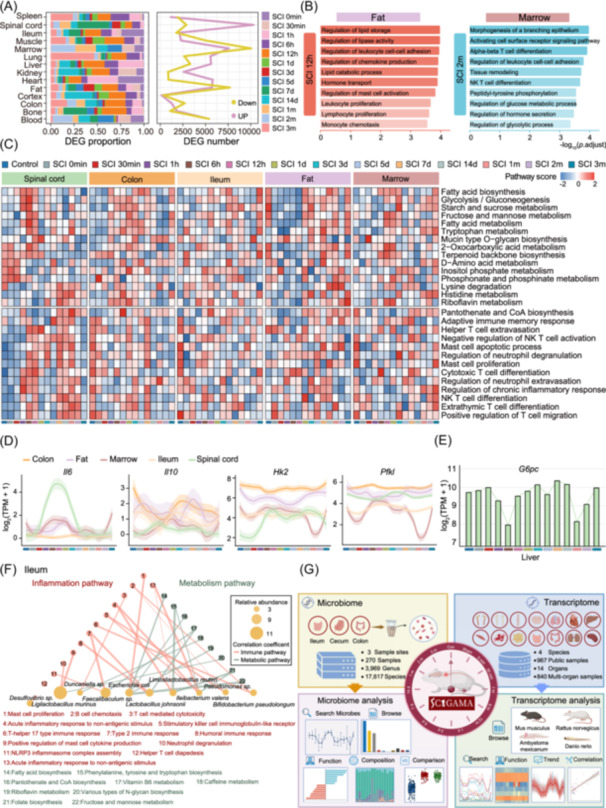
Integrated metagenomic and transcriptomic analyses unravel organ‐ and time‐specific immune and metabolic reprogramming in multiple organs post‐SCI. (A) Bar graph (left *x*‐axis, showing percentage of differentially expressed genes (DEGs)) and line graph (right *x*‐axis, showing total number of DEGs) illustrating DEGs in various organs at different time points post‐SCI, relative to Control group. (B) GO enrichment of upregulated genes in adipose tissue and bone marrow. (C) Heatmap illustrating the organ‐ and time‐specific alterations in immune and metabolic pathways. (D) Line plots of expression levels of *Il6*, *Il10*, *Hk2* and *Pfkl* in various organs. (E) Bar graph showing the expression level of *G6pc* in the liver. (F) Network visualization of correlations between dominant ileal microbial taxa and host inflammatory and metabolic pathways. (G) Schematic of SCIGAMA.

To understand the systemic impact of SCI, we investigated transcriptomic alterations in peripheral tissues, among which adipose tissue and bone marrow exhibited the most profound perturbations (Figure 2A). Gene Ontology (GO) enrichment analysis revealed that adipose tissue was enriched in pathways related to lipid storage and leukocyte adhesion, while bone marrow showed enrichment in glucose metabolism and leukocyte adhesion (Figure 2B). We further employed Gene Set Variation Analysis to explore immune and metabolic pathway regulation across organs. This revealed organ‐specific transcriptional trajectories, with marked activation of riboflavin metabolism and glycolysis in the colon and ileum during the acute phase, while the spinal cord, fat, and heart exhibited distinct responses (Figure 2C, Figure S3A). Inflammatory pathways were particularly heterogeneous; the spinal cord maintained sustained inflammation into the chronic phase, whereas the colon showed earlier resolution, with substantial reductions by 7 days post‐SCI (Figure 2C). Notably, T cell migration pathways remained activated in the spinal cord during the chronic phase, but transiently upregulated in the liver and heart, returning to baseline in the chronic phase (Figure S3A).

We investigated changes in key inflammatory cytokines. In the spinal cord, *Il6* expression peaked at 6 h (Log_2_FC = 7.04, *p*. adj < 0.01) after injury and then gradually declined. However, *Il6* in the fat displayed a more fluctuating pattern, reaching a nadir at 5 days (Log_2_FC = −4.23, *p*. adj < 0.01). Together, these findings indicated that inflammatory responses exhibit marked organ‐specific temporal heterogeneity. *Il10* exhibited a distinct triphasic pattern, which was particularly evident in the colon. For instance, its expression initially declined at 12 h (Log_2_FC = −2.57, *p*. adj < 0.01), rebounded significantly by 14 days (Log_2_FC = −1.12, *p*. adj < 0.05), and subsequently decreased again during the chronic phase at 3 months (Log_2_FC = −1.98, *p*. adj < 0.01) (Figure 2D). Other key factors, including *Ccl2*, *Ccl5*, *Il1a*, *Il1b*, *Ifng*, and *Tnf*, exhibited dynamic changes across multiple organs (Figure S3B). Notably, colonic *Ifng* displayed early suppression followed by a rebound, reflecting immune quiescence and subsequent adaptive remodeling following SCI. Metabolically, genes encoding glycolytic enzymes, *Hk2* and *Pfkl,* were upregulated with organ‐specific kinetics (Figure 2D). While *G6pc* expression in the liver decreased initially and then gradually recovered (Figure 2E). This suggests metabolic interplay, where lactate and glycolytic byproducts are shuttled to the liver for gluconeogenesis, contributing to systemic glucose homeostasis. Collectively, SCI appears to induce systemic metabolic reprogramming, akin to the Cori cycle, with increased glycolysis in peripheral tissues and concurrent adaptation in the liver.

The gut microbiota exhibits synchronous dynamic associations with distal organ functions. The gut serves as the primary interface for host‐microbe interactions. We further investigated the interplay between gut microbial taxa and functional pathways in the ileum and colon following SCI. In the ileum, *Ligilactobacillus murinus* exhibited associations with cytokine production and neutrophil degranulation, whereas *Escherichia coli* was linked to metabolic pathways, including fatty acid biosynthesis and CoA biosynthesis (Figure 2F). In the colon, *Ligilactobacillus murinus* was associated with chronic inflammatory responses to non‐antigenic stimuli, while *Acetatifactor* sp. showed broad associations with immune and metabolic pathways (Figure S3C). Temporal analysis revealed that *Bifidobacterium pseudolongum, Lactobacillus johnsonii*, and *Faecalibaculum* sp. were enriched in the ileum during the acute phase, while *Pseudomonas* sp. and *Desulfovibrio* sp. predominated in the chronic phase. In the colon, *Bacteroides* sp. and *Duncaniella* sp. peaked during the subacute phase (Figure S3D). Furthermore, ileal *Escherichia coli* exhibited extensive correlations with multiple organs, suggesting systemic influence beyond the gut (Figure S3E–G).

To consolidate and translate our multi‐omics findings into an accessible resource, we developed the SCIGAMA (Figure 2G). This platform contains in‐house and public sequencing data, supporting the investigation of systemic SCI responses (Figure S4A,B), including 2077 samples: 270 gut metagenomic, 840 multi‐organ transcriptomic, and 967 public samples (Figure S4C–F). It supports three modules: (a) temporal and spatial dynamics, which explore microbial diversity and functional enrichment; (b) multi‐organ transcriptomic profiling, which visualizes organ‐ and time‐specific gene expression; (c) integrated correlation analysis, which performs differential expression and microbiota‐transcriptome correlation analyses. SCIGAMA provides an intuitive web‐based interface, enabling hypothesis generation and comparative omics research, offering a systems‐level perspective on SCI for biomarker and therapeutic development.

SCI is a dynamic process, characterized by evolving molecular, cellular, and systemic alterations. The intricate interplay between the gut microbiota and host physiology represents a critical, yet often underappreciated, dimension that is closely synchronized with both local spinal cord pathology and systemic multi‐organ dysfunction following SCI. We identify microbiome shock, an acute dysbiosis following SCI, characterized by loss of beneficial commensals and expansion of opportunistic pathogens, with incomplete recovery. Defining its spatiotemporal dynamics enables the identification of phase‐specific intervention windows. Although mechanisms remain unclear, microbiome shock likely results from SCI‐induced gut dysmotility and autonomic dysfunction [17], coupled with systemic stress responses and immune dysregulation [18] that further disrupt host‐microbe homeostasis and promote opportunistic taxa expansion.

Systemic multi‐organ responses to SCI significantly impact long‐term survival, with many patients dying from complications in other organs [19]. Our multi‐organ transcriptomic analysis revealed distinct, organ‐specific temporal patterns of pathway activation during the chronic phase, underscoring the necessity for tailored, spatiotemporally precise therapeutic strategies. Broad anti‐inflammatory strategies may have harmful effects, necessitating organ‐specific, timed interventions, particularly for life‐sustaining organs like the heart and lungs, to improve both survival and quality of life in SCI patients.

Although stage‐specific interventions show promise in mouse models, their translation into clinical practice faces several challenges. However, the pathophysiological phases of human SCI align with animal models, providing a temporal framework for stage‐specific treatments [20]. Building on this commonality, targeted treatments could be implemented during critical phases of human SCI, such as the acute or subacute stage. For instance, probiotics that are depleted during specific phases could be supplemented, pathogenic bacteria selectively suppressed, or stage‐specific genes modulated to promote spinal cord repair and multi‐organ functional recovery.

This study has several limitations. The murine model may not fully recapitulate the complexity of human gut microbiota diversity or multi‐organ responses to SCI. Additionally, only female mice were included to reduce variability, and future studies should include both sexes. Furthermore, the correlations observed between specific microbial taxa and host transcriptomic pathways must be interpreted with caution. While our correlation networks serve as a robust systems‐level hypothesis‐generating resource, establishing definitive causal relationships will require future validation using gnotobiotic models or targeted interventions. Finally, public datasets in SCIGAMA were uniformly processed but excluded from core analyses due to heterogeneity, serving instead as an exploratory resource. Nevertheless, this work provides a systematic foundational resource and conceptual framework highlighting the importance of microbial and systemic homeostasis in SCI recovery.

In summary, we identify microbiome shock as an early event after SCI, marked by acute dysbiosis and microbial shifts. Multi‐organ transcriptomic analyses reveal highly coordinated associations linking gut microbiota fluctuations to transcriptomic reprogramming in distant organs, underscoring the systemic footprint of SCI. We further present SCIGAMA, an open‐access interactive resource to facilitate future mechanistic and therapeutic studies.

## AUTHOR CONTRIBUTIONS

**Chi Zhang**: Writing—original draft; formal analysis; data curation; conceptualization; methodology. **Yufei Du**: Writing—original draft; data curation; formal analysis; investigation; visualization; software; methodology. **Mingxin Wu**: Writing—original draft; Visualization; software; formal analysis; investigation. **Chuang Li**: Software; methodology; data curation; visualization. **Ruizhi Jiang**: Investigation; formal analysis. **Enlin Qi**: Methodology; data curation. **Shaolong Li**: Investigation; methodology. **Xianfu Yi**: Writing—review and editing; supervision; project administration; conceptualization. **Bo Chu**: Writing—review and editing; supervision; validation; resources. **Shiqing Feng**: Writing—review and editing; project administration; funding acquisition. **Hengxing Zhou**: Writing—review and editing; project administration; resources; supervision; conceptualization; funding acquisition. All authors have read the final manuscript and approved it for publication.

## CONFLICT OF INTEREST STATEMENT

The authors declare no conflicts of interest.

## ETHICS STATEMENT

The studies involving animals were approved by the Medical Ethics Committee of Qilu Hospital of Shandong University (No. DWLL‐2021061).

## Supporting information


**Figure S1:** Temporal changes in microbial diversity and differential abundance post‐SCI.
**Figure S2:** Temporal changes in microbial abundance and specific microbial functions.
**Figure S3:** Gut microbiota composition and function are associated with systemic metabolic and immune alterations following spinal cord injury.
**Figure S4:** SCIGAMA: a robust multi‐omics data processing pipeline and integrated resource for spinal cord injury studies.

**Table S1:** Differential microbiota at the phylum level in different regions following spinal cord injury.
**Table S2:** Functional enrichment of microbes from different gut sites.
**Table S3:** The datasets included in SCIGAMA.

## Data Availability

Research data are not shared. The SCIGAMA website is available at http://58.247.16.28:30080/SCIGAMA/index/homePage. The SCIGAMA database will be maintained and regularly updated by our research team to ensure its continued accessibility. For usage instructions and additional details, please refer to the SCIGAMA Help page (http://58.247.16.28:30080/SCIGAMA/help/1/helpPage). The data and figure‐related codes are stored at https://github.com/Magetutor/Microbiome-shock-in-spinal-cord-injury. Supplementary materials (methods, figures, tables, graphical abstract, slides, videos, Chinese translated version, and updated materials) can be found in the online DOI or iMeta Science http://www.imeta.science/.
