# Supplementary material for: Spinal cord injury induces acute microbiome shock and system‐wide transcriptomic reprogramming
Source: Imeta. 2026 May 3;5(2):e70128. doi: 10.1002/imt2.70128 (PMC13147949; doi:10.1002/imt2.70128)
Supplement: Supplementary file 1 — Figure S1: Temporal changes in microbial diversity and differential abundance post‐SCI. Figure S2: Temporal changes in microbial abundance and specific microbial functions. Figure S3: Gut microbiota composition and function are associated with systemic metabolic and immune alterations following spinal cord injury. Figure S4: SCIGAMA: a robust multi‐omics data processing pipeline and integrated resource for spinal cord injury studies. [file IMT2-5-e70128-s002.docx]

**Supporting information to**

**Spinal cord injury induces acute microbiome shock and system-wide transcriptomic reprogramming**

**Running title**: Spinal cord injury induces microbiome shock and systemic reprogramming

Chi Zhang^1#^, Yufei Du^2#^, Mingxin Wu^3#^, Chuang Li^1#^, Ruizhi Jiang^4^, Enlin Qi^4^, Shaolong Li^4^, Xianfu Yi^5^*****, Bo Chu^6^*****, Shiqing Feng^1, 4^***** and Hengxing Zhou ^4^*****

^1^Department of Orthopaedics, The Second Qilu Hospital of Shandong University, Shandong University Centre for Orthopaedics, Cheeloo College of Medicine, Shandong University, Jinan 250033, China.

^2^Department of Endocrinology and Metabolism, The Second Qilu Hospital of Shandong University, Cheeloo College of Medicine, Shandong University, Jinan 250033, China.

^3^Department of Spine Osteopathia, The First Affiliated Hospital of Guangxi Medical University, Nanning 530021, China.

^4^Department of Orthopaedics, Qilu Hospital of Shandong University, Shandong University Centre for Orthopaedics, Advanced Medical Research Institute, Cheeloo College of Medicine, Shandong University, Jinan 250012, China.

^5^Department of Bioinformatics, School of Basic Medical Sciences, Tianjin Medical University, Tianjin 300070, China.

^6^Department of Cell Biology, School of Basic Medical Sciences, Cheeloo College of Medicine, Shandong University, Jinan 250012, China.

#These authors contributed equally: Chi Zhang, Yufei Du, Mingxin Wu and Chuang Li

*Correspondence: [zhouhengxing@sdu.edu.cn](mailto:xxx@edu.cn) (Hengxing Zhou); shiqingfeng@sdu.edu.cn (Shiqing Feng); chubo123@sdu.edu.cn (Bo Chu); yixianfu@tmu.edu.cn (Xianfu Yi)

**Supplementary methods**

**Mouse model of spinal cord injury**

Female C57BL/6 mice (8–10 weeks old) were obtained from the SPF (Beijing) Biotechnology Co., Ltd, China. The exclusive use of female mice in this study was based on considerations of post-operative care feasibility and experimental reproducibility. While prior studies indicate that sex may influence pathological progression and functional recovery after spinal cord injury (SCI), we utilized a single sex to control for potential confounding variables, such as hormonal fluctuations. Future investigations will incorporate male mouse models to systematically evaluate the impact of sex differences on the findings. The SCI model was established using a previously described methods [1]. All surgical procedures were performed under sterile conditions in a temperature-controlled operating room. Mice were anesthetized with isoflurane (RWD, R510-22, Guangdong, China). Following a midline incision at the T8 vertebral level, muscles were carefully dissected to expose the T7-T9 spinous processes. The T7 and T9 spinous processes were stabilized with fixators, and a T8 laminectomy was performed to fully expose the spinal cord. A moderate spinal cord contusion was induced using the MASCIS impactor model III (WM Keck, USA) with a 5 g weight and a 12.5 mm impact area. For the Sham group, mice underwent identical surgical procedures including anesthesia, incision, and T8 laminectomy, but did not receive the contusive impact. Following the contusion, the muscles, fascia, and skin were sutured gently. The studies involving animals were approved by Medical Ethics Committee of Qilu Hospital of Shandong University (IACUC Issue NO. DWLL- 20210061).

**Animal housing and perioperative management**

All mice were housed under specific pathogen-free (SPF) conditions in a controlled environment (12-hour light/dark cycle, 20–25°C, 40–60% humidity) with ad libitum access to autoclaved water and standard chow. To minimize potential cage effects on the gut microbiome, mice were co-housed (5 mice per cage) for at least one week prior to surgery and then randomly assigned to the Sham or SCI groups. Bedding was changed weekly with autoclaved materials. Subcutaneous injection of buprenorphine sustained-release (0.1 mg/kg) for perioperative analgesia. Bladder evacuation was performed twice daily for the first 7 days post-SCI, until spontaneous urination resumed. No antibiotics or analgesics were administered at any time post-SCI to avoid confounding effects on the native gut microbiome. These standardized housing and management protocols were implemented to ensure consistency and to minimize non-experimental variables that could influence the gut microbiome and systemic transcriptome analyses.

**Tissue perfusion and collection**

Tissues from mice were collected at different stages, including control, Sham, and 13 distinct time points post-SCI (0 minutes, 30 minutes, 1 hour, 6 hours, 12 hours, 1 day, 3 days, 5 days, 7 days, 14 days, 1 month, 2 months, and 3 months), This design spans the acute, subacute, and chronic pathophysiological phases of SCI and aligns with time points commonly used in related studies [2−6]. For the sham group, tissues were collected immediately after the sham surgery procedure. Given that gut microbiome changes observed between mice of different ages are not primarily driven by aging itself [7], a separate sham group was not included at each longitudinal time point. For gut microbiome analysis, biological replicates (n = 6) were collected per group. For multi-organ transcriptome profiling, biological replicates (n = 4) were collected per group. Following anesthesia, blood samples was collected via the jugular vein and stored at -80°C. An incision was made along the rib cage on both sides of the thorax and abdomen, extending from the xiphoid process. The abdominal skin then dissected to comprehensively expose the intestines. Contents from the ileum, cecum, and colon were collected, quickly frozen in liquid nitrogen, and subsequently stored at -80°C until further analysis.

The spleen was excised using surgical scissors and immediately frozen in liquid nitrogen, followed by tissue perfusion. Mice were perfused transcardially with ice-cold phosphate-buffered saline (PBS) until the effluent from the right atrium was clear and the liver appeared uniformly pale. Subsequently, the cerebral cortex, lungs, heart, liver, kidneys, spinal cord, adipose tissue, colon, small intestine, muscle, and bone were rapidly harvested and snap-frozen in liquid nitrogen. Prior to freezing, adipose tissue, colon, small intestine, muscle, and bone tissues were rinsed with PBS. The femurs were excised from the hind limbs of the mice, and muscle tissue attached to the femur was removed using gauze. The epiphyses at both ends of the femur were clipped, and the bone marrow cavity was rinsed with PBS. The PBS flush was collected, filtered to remove impurities, and centrifuged. Finally, the resulting cell suspension was aliquoted into low-temperature storage tubes in liquid nitrogen.

**Microbiome library**

Genomic DNA was extracted from 100–200 mg of mouse gut contents using the FastPure Stool DNA Isolation Kit (MJYH, Shanghai, China), with sterile water serving as a negative control. DNA quality was assessed using a 1% agarose gel electrophoresis, and its concentration and purity were determined with a NanoDrop 2000 spectrophotometer (Thermo Scientific, USA). The DNA was fragmented to an average size of ~350 bp using the Covaris M220 (Gene Company, China), and paired-end libraries were constructed with the NEXTFLEX Rapid DNA-Seq kit (Bioo Scientific, USA). Sequencing was performed on the Illumina NovaSeq™ X Plus platform (Illumina, USA) by Majorbio Bio-Pharm Technology Co. (Shanghai, China) using the NovaSeq X Series 25B Reagent Kit.

**Microbiome analysis**

Raw reads were processed to remove adapters and low-quality sequences (length < 50 bp or average quality < 20) using Fastp (v0.2 0.0) [8]. Reads were aligned to the mouse genome using BWA(v0.7.17) [9] and any hit associated with the reads and their mated reads were removed. Assemblies were generated with MEGAHIT(v1.1.2) [10], selecting contigs ≥ 300 bp. A non-redundant gene catalog was constructed with CD-HIT (v4.7) [11], and gene abundance was estimated using SOAPaligner (v2.21) [12].

Taxonomic classification and functional annotation were performed using DIAMOND (v2.0.13) [13] against the NCBI NR database. KEGG functional annotation was based on comparisons of gene sequences to the KEGG database. Differential abundance analysis at the taxonomic, functional, and gene levels was conducted using Kruskal-Wallis tests. P-values from differential analyses were adjusted for multiple testing using the Benjamini-Hochberg false discovery rate (FDR) correction, and an adjusted p-value threshold of < 0.05 was considered statistically significant. Alpha diversity was assessed by calculating Shannon, Simpson, and Chao indices for each sample, and results are presented as box plots. For beta diversity analysis, principal coordinate analysis (PCoA), non-metric multidimensional scaling (NMDS), and ANOSIM tests were performed. The linear discriminant analysis effect size (LEfSe) method was used to identify microbial taxa significantly differing between groups, with an LDA score threshold of > 2. To ensure statistical robustness against the zero-inflated nature of microbiome data, differential abundance analysis at the taxonomic was rigorously evaluated using a one-way permutation test (Monte Carlo approximation with 9,999 resamples) via the coin (v1.4-3) R package. P-values from differential analyses were adjusted for multiple testing using the Benjamini-Hochberg false discovery rate (FDR) correction, and an adjusted p-value threshold of < 0.05 was considered statistically significant.

**Self-generated RNA sequencing (RNA-seq) libraries**

Total RNA was extracted using the Total RNA Extraction Kit (Solarbio, R1200). Ribosomal RNA (rRNA) was depleted from the total RNA using the Ribo-Zero rRNA Removal Kit (Illumina, USA) according to the manufacturer’s instructions. Stranded RNA sequencing libraries were then constructed from the rRNA-depleted RNA using the TruSeq Stranded Total RNA Library Prep Kit (Illumina, USA) following the manufacturer’s protocol. Library quality and quantity were assessed using the BioAnalyzer 2100 system (Agilent Technologies, USA). The qualified libraries (10 pM) were denatured into single-stranded DNA molecules, captured on Illumina flow cells, amplified in situ into clusters, and subjected to paired-end sequencing (2 × 150 bp) on an Illumina HiSeq 4000 instrument as per the manufacturer’s guidelines.

**Public data collection**

Microarray and RNA sequencing raw data were retrieved from the NCBI Gene Expression Omnibus (GEO) database (https://www.ncbi.nlm.nih.gov/geo/) in December 2024. Employing 'spinal cord injury' as the search term, data were selected for 4 species with available datasets: *Ambystoma mexicanum*, *Danio rerio*, *Mus musculus*, and *Rattus norvegicus*. Out of the 194 publicly available datasets identified. Following the exclusion of data pertaining to gene modifications, materials, and other forms of sequencing (including scRNA-Seq, ChIP-Seq, miRNA-Seq, ncRNA-Seq, snRNA-seq, and ATAC-Seq), a total of 55 data points were included in the subsequent analysis.

It is important to note that these public datasets originate from diverse independent studies, which introduce inherent heterogeneity in factors such as animal strain, sex, age, housing conditions, injury models, and experimental protocols. To ensure comparability at the basic processing level, all raw data were uniformly processed using the standardized pipeline described below (Table S3 and Transcriptome Analysis section). However, due to the significant confounding factors mentioned, the public data were not directly integrated with our self-generated data for combined analysis. Instead, they serve as a supplementary resource within the SCIGAMA platform to provide broader context.

The raw data were subsequently processed following the procedures outlined below (Table S3).

**Transcriptome analysis**

The quality of paired-end reads was assessed using FastQC (v0.11.9), followed by trimming of 3’ adapters and removal of low-quality reads with Cutadapt (v1.9.3). High-quality reads were then aligned to the mouse reference genome (mm10) using HISAT2 (v2.0.4). Genes with counts of less than one per million in all samples were excluded from downstream analyses. Gene expression levels were estimated using the GENCODE (GRCm38.p6) GTF gene annotation file, with expression quantified in Transcripts Per Million (TPM) per kilobase of transcript.

Gene expression levels were estimated, and sequencing depth was normalized using the TPM (Transcripts Per Million) method. Differentially expressed genes (DEGs) were identified using the limma (v2.9.8) and DESeq2 (v1.4.5) packages. For DE analysis, each organ was analyzed separately using a time-specific design matrix (~ 0 + time, where 'time' represents the specific time point) to compare each post-SCI time point against the control group. *P*-values were adjusted for multiple testing using the Benjamini-Hochberg method, and genes exhibiting a fold change ≥ 1 and an adjusted *P*-value ≤ 0.05 were considered differentially expressed. Functional annotation was performed using the clusterProfiler (v4.4.0) R package, including Gene Ontology (GO) and Kyoto Encyclopedia of Genes and Genomes (KEGG) pathway enrichment analysis. Statistical significance for GO and KEGG enrichment was determined using the hypergeometric test, with *p*-values adjusted using the Benjamini-Hochberg FDR method (adjusted *p*-value ≤ 0.05).

For time-series gene clustering analysis, fuzzy c-means clustering was conducted using the Mfuzz (v2.68.0) R package, with the number of clusters set to 8 and the fuzziness coefficient set to 1.5. which were then integrated with enrichment analysis results. Cluster visualization was achieved using the ClusterGVis (v0.1.2) R package, which generating heatmaps and combining them with enrichment analysis results. Gene correlation analysis was conducted using the “cor.test” function to compute Pearson correlation coefficients, enabling visualization of correlations between specific genes or gene pairs.

To further investigate pathway-level changes, gene set variation analysis (GSVA, v1.46.0) was employed to quantify pathway activity scores for the 15 experimental groups across the 14 organs examined. GSVA, a non-parametric, unsupervised method, was utilized to assess the relative enrichment of predefined gene sets derived from the GO and KEGG databases within each sample. This approach transforms gene expression data into pathway activity scores, thereby facilitating the visualization of pathway dynamics across different tissues and time points, without performing formal statistical hypothesis testing on these scores.

**REFERENCES**

1. Liu, Kai, Yi Lu, Jae K. Lee, Ramsey Samara, Rafer Willenberg, Ilse Sears-Kraxberger, Andrea Tedeschi, et al. 2010. “PTEN deletion enhances the regenerative ability of adult corticospinal neurons.” *Nature Neuroscience* 13: 1075–1081. https://doi.org/10.1038/nn.2603

2. Amo-Aparicio, Jesus, Alba Sanchez-Fernandez, Suzhao Li, Elan Z. Eisenmesser, Cecilia Garlanda, Charles A. Dinarello, Ruben Lopez-Vales. 2021. “Extracellular and nuclear roles of IL-37 after spinal cord injury.” *Brain, Behavior, and Immunity* 91: 194–201. https://doi.org/10.1016/j.bbi.2020.09.026

3. Amo-Aparicio, Jesus, Joana Garcia-Garcia, Isaac Francos-Quijorna, Andrea Urpi, Anna Esteve-Codina, Marta Gut, Albert Quintana, Ruben Lopez-Vales. 2021. “Interleukin-4 and interleukin-13 induce different metabolic profiles in microglia and macrophages that relate with divergent outcomes after spinal cord injury.” *Theranostics* 11: 9805–9820. https://doi.org/10.7150/thno.65203

4. Tan, Zijian, Shangyao Qin, Yimin Yuan, Xin Hu, Xiao Huang, Hong Liu, Yingyan Pu, Cheng He, Zhida Su. 2022. “NOTCH1 signaling regulates the latent neurogenic program in adult reactive astrocytes after spinal cord injury.” *Theranostics* 12: 4548–4563. https://doi.org/10.7150/thno.71378

5. Shu, Muya, Xiaoyu Xue, Hu Nie, Xianming Wu, Minghan Sun, Lianyong Qiao, Xing Li, et al. 2022. “Single-cell RNA sequencing reveals Nestin+ active neural stem cells outside the central canal after spinal cord injury.” *Science China Life Sciences* 65: 295–308. https://doi.org/10.1007/s11427-020-1930-0

6. Xue, Xiaoyu, Muya Shu, Zhifeng Xiao, Yannan Zhao, Xing Li, Haipeng Zhang, Yongheng Fan, et al. 2022. “Lineage tracing reveals the origin of Nestin-positive cells are heterogeneous and rarely from ependymal cells after spinal cord injury.” *Science China Life Sciences* 65: 757–769. https://doi.org/10.1007/s11427-020-1901-4

7. Litichevskiy, Lev, Maya Considine, Jasleen Gill, Vasuprada Shandar, Timothy O. Cox, Hélène C. Descamps, Kevin M. Wright, et al. 2025. “Gut metagenomes reveal interactions between dietary restriction, ageing and the microbiome in genetically diverse mice.” *Nature Microbiology* 10: 1240–1257. https://doi.org/10.1038/s41564-025-01963-3

8. Chen, Shifu, Yanqing Zhou, Yaru Chen, Jia Gu. 2018. “fastp: an ultra-fast all-in-one FASTQ preprocessor.” *Bioinformatics* 34: i884–i890. https://doi.org/10.1093/bioinformatics/bty560

9. Li, Heng, Richard Durbin. 2009. “Fast and accurate short read alignment with Burrows–Wheeler transform.” *Bioinformatics* 25: 1754–1760. https://doi.org/10.1093/bioinformatics/btp324

10. Li, Dinghua, Chi-Man Liu, Ruibang Luo, Kunihiko Sadakane, Tak-Wah Lam. 2015. “MEGAHIT: an ultra-fast single-node solution for large and complex metagenomics assembly via succinct de Bruijn graph.” *Bioinformatics* 31: 1674–1676. https://doi.org/10.1093/bioinformatics/btv033

11. Fu, Limin, Beifang Niu, Zhengwei Zhu, Sitao Wu, Weizhong Li. 2012. “CD-HIT: accelerated for clustering the next-generation sequencing data.” *Bioinformatics* 28: 3150–3152. https://doi.org/10.1093/bioinformatics/bts565

12. Li, Ruiqiang, Yingrui Li, Karsten Kristiansen, Jun Wang. 2008. “SOAP: short oligonucleotide alignment program.” *Bioinformatics* 24: 713–714. https://doi.org/10.1093/bioinformatics/btn025

13. Buchfink, Benjamin, Chao Xie, Daniel H. Huson. 2015. “Fast and sensitive protein alignment using DIAMOND.” *Nature Methods* 12: 59–60. https://doi.org/10.1038/nmeth.3176

**Supplementary figures**


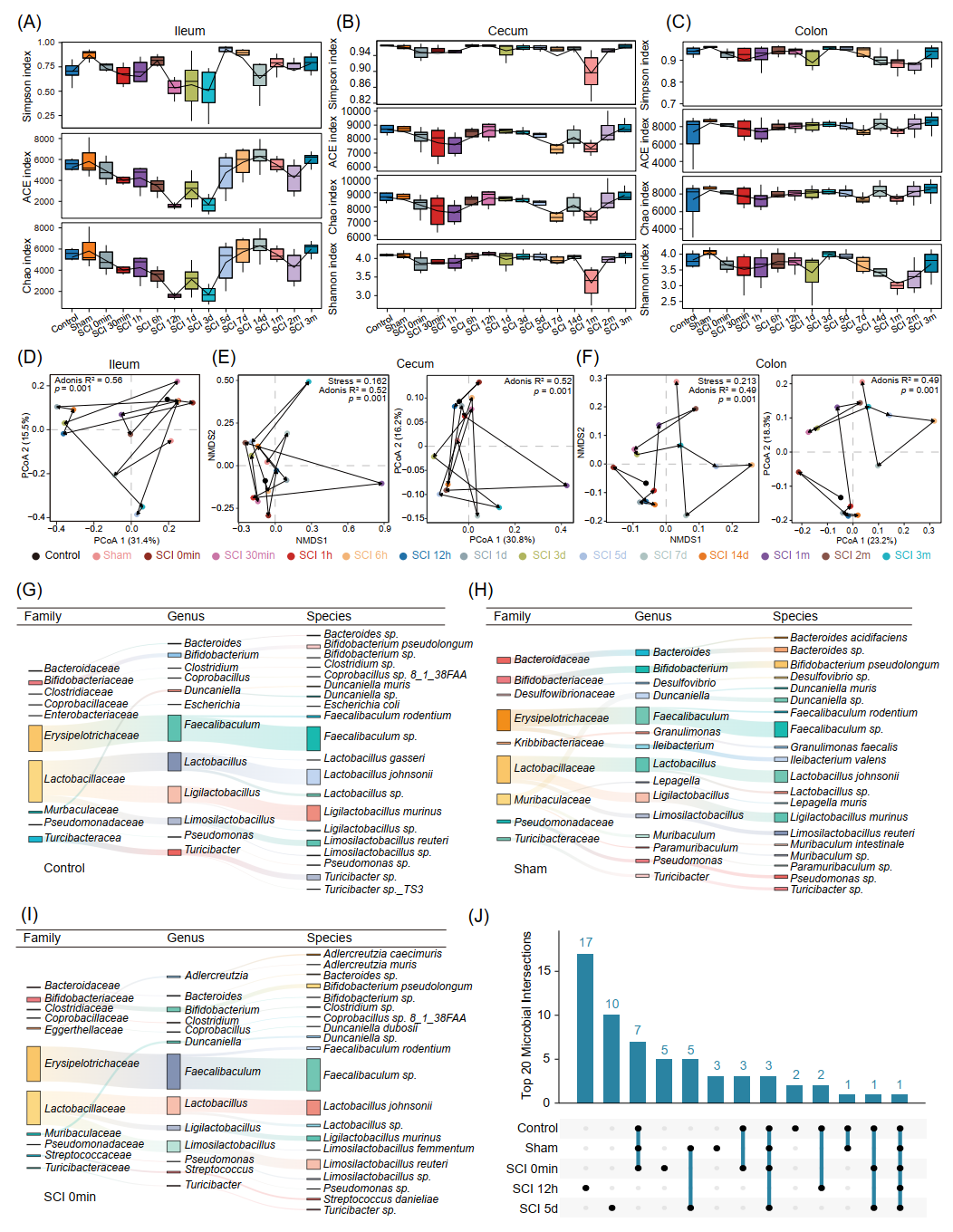


**Figure S1 Temporal changes in microbial diversity and differential abundance post spinal cord injury.** (A) α-diversity in ileum microbiota at different time points post spinal cord injury (SCI) using Chao index, ACE index, and Simpson index; (B) α-diversity in cecum microbiota at different time points post-SCI using Chao index, ACE index, Simpson index, and Shannon index; (C) α-diversity in colon microbiota at different time points post SCI using Chao index, ACE index, Simpson index, and Shannon index; (D) β-diversity in ileum microbiota at different time points post SCI using principal coordinates analysis (PCoA); (E) β-diversity in cecum microbiota at different time points post SCI using non-metric multidimensional scaling (NMDS) and PCoA; (F) β-diversity in colon microbiota at different time points post-SCI using NMDS and PCoA; (G-I) Hierarchical structure of intestinal microbiota changes in Control, Sham, and SCI 0min groups, shown by Sankey plots from family to genus to species level; (J) Overlap of the top 20 differential microbiota in Control, Sham, SCI 0min, SCI 12h, and SCI 5d groups.


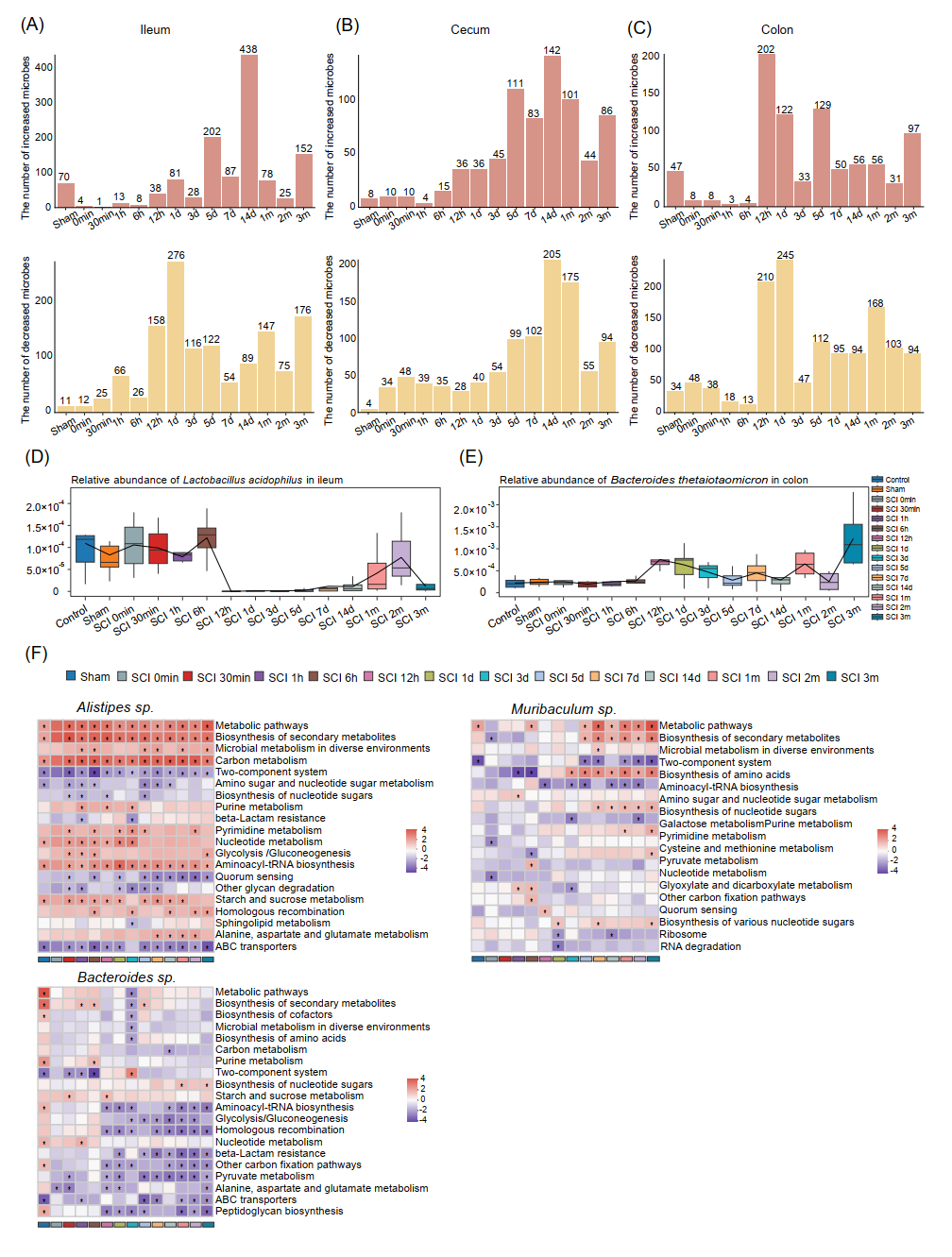


**Figure S2 Temporal changes in microbial abundance and specific microbial functions.** (A-C) The number of microorganisms with increased or decreased abundance in the ileum (A), cecum (B), and colon (C) microbiota compared to the Control group; (D and E) Box plots depicting the relative abundance of *Lactobacillus acidophilus* (D) and *Bacteroides thetaiotaomicron* (E) across different time points post-SCI; （F）Functional annotation analysis results for microbial taxa that remained unclassified at the genus or species level.


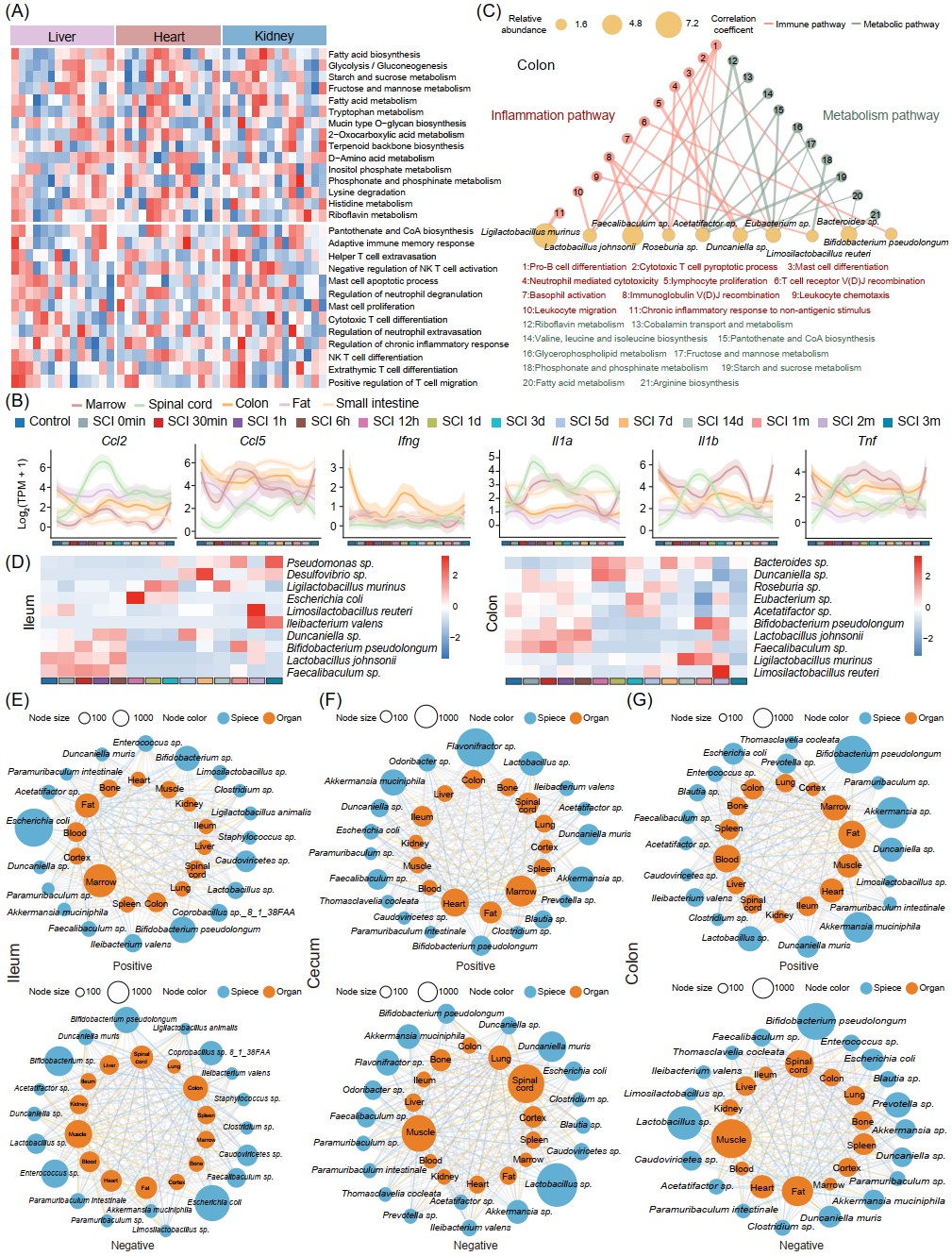


**Figure S3 Gut microbiota composition and function are associated with systemic metabolic and immune alterations following SCI.** (A) Heatmap of metabolic and immune pathway scores in the liver, heart, and kidney across various time points post-SCI. (B) Line plots depicting the relative mRNA expression levels of cytokines *Ccl2*, *Ccl5*, *Ifng*, *Il1a*, *Il1b*, and *Tnfa* in various organs at different time points post-SCI. (C) Correlation network between the top 10 most abundant microorganisms in the colon and immune-metabolic functions. (D) Heatmap showing the abundance of the respective top 10 microorganisms in the ileum and colon. (E-G) Correlation network between the top 20 most abundant microorganisms across three intestinal segments and gene expression in various organs.


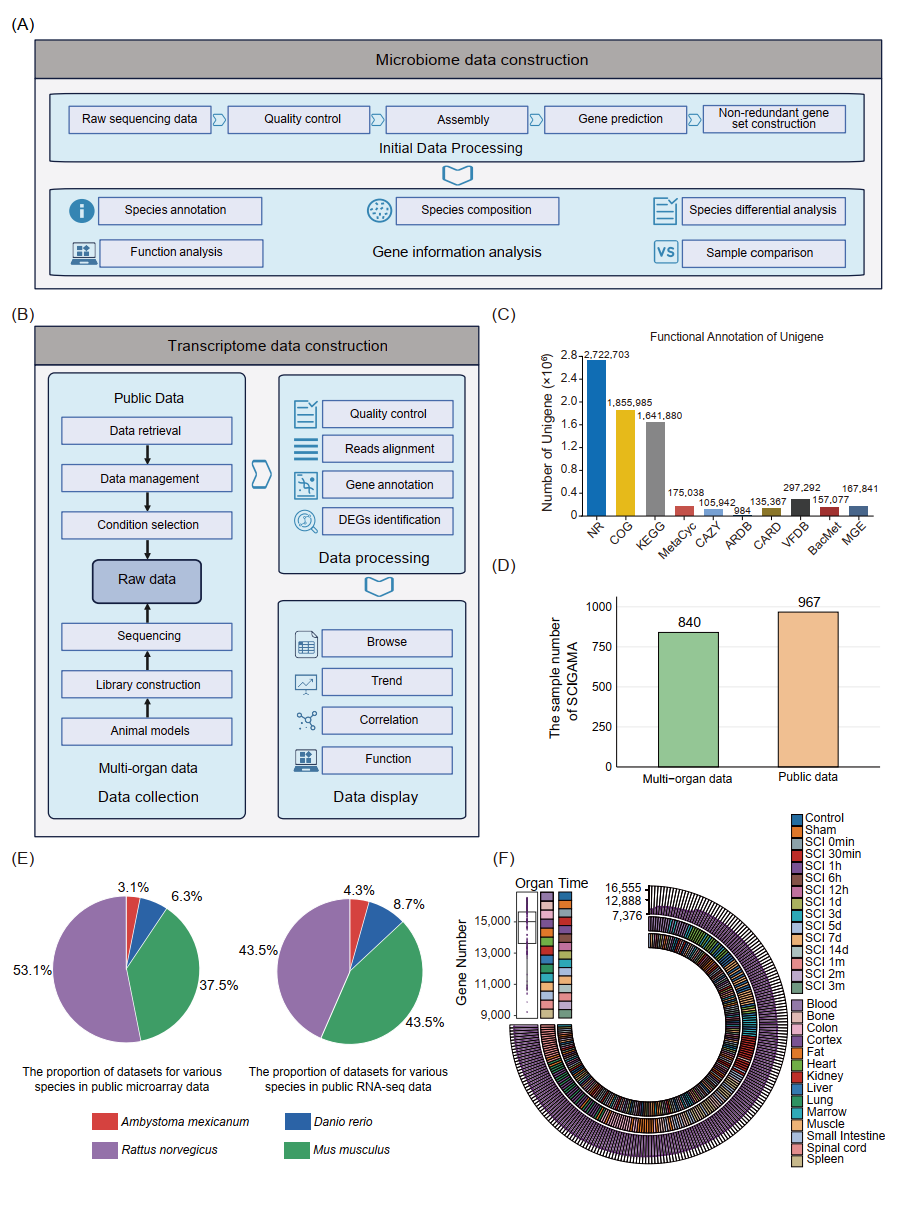


**Figure S4 SCIGAMA: A robust multi-omics data processing pipeline and integrated resource for spinal cord injury studies.** (A) Workflow for microbiome data processing. (B) Workflow for transcriptome data processing of public and proprietary RNA-seq datasets. (C) Unique gene counts annotated by different databases; y-axis indicates the number of unique genes (in millions, ×10^6^). (D) Number of public datasets and multi-organ transcriptome datasets incorporated into SCIGAMA. (E) Species composition of publicly available microarray and RNA-seq datasets. (F) Circular plot showing the number of expressed genes across 14 organs and 15 time points.
